# Responding to CheckCIF “Experts”

**DOI:** 10.1063/4.0000889

**Published:** 2025-10-27

**Authors:** Carla Slebodnick, Abigail Edwards, Landon J. Elkins, Tegan A. Makal

**Affiliations:** 1 Department of Chemistry, Virginia Tech, Blacksburg, VA, USA; 2 Department of Natural Sciences, The University of Virginia's College at Wise, Wise, VA, USA

## Abstract

In response to a manuscript submission, “Referee 1” provided the following evaluation:

“I recommend “Major Revision” or "Reject and Resubmit", considering the quality of the revised manuscript. I have serious concerns as listed below.

Additional processing and analysis should be conducted to ensure that at least alerts A and B do not occur in CheckCIF. For samples with high R- values (Cu2BADI_DMF), detailed discussions about the structure should be avoided.

Samples with inconsistent elemental analysis results should be reanalyzed.”

The structures of concern will be presented,^1^ along with discussion of how to respond to such reviews.
